# Ultrathin high band gap solar cells with improved efficiencies from the world’s oldest photovoltaic material

**DOI:** 10.1038/s41467-017-00582-9

**Published:** 2017-09-25

**Authors:** Teodor K. Todorov, Saurabh Singh, Douglas M. Bishop, Oki Gunawan, Yun Seog Lee, Talia S. Gershon, Kevin W. Brew, Priscilla D. Antunez, Richard Haight

**Affiliations:** IBM Thomas J. Watson Research Center, 1101 Kitchawan Road, Yorktown Heights, NY 10598 USA

## Abstract

Selenium was used in the first solid state solar cell in 1883 and gave early insights into the photoelectric effect that inspired Einstein’s Nobel Prize work; however, the latest efficiency milestone of 5.0% was more than 30 years ago. The recent surge of interest towards high-band gap absorbers for tandem applications led us to reconsider this attractive 1.95 eV material. Here, we show completely redesigned selenium devices with improved back and front interfaces optimized through combinatorial studies and demonstrate record open-circuit voltage (*V*_OC_) of 970 mV and efficiency of 6.5% under 1 Sun. In addition, Se devices are air-stable, non-toxic, and extremely simple to fabricate. The absorber layer is only 100 nm thick, and can be processed at 200 ˚C, allowing temperature compatibility with most bottom substrates or sub-cells. We analyze device limitations and find significant potential for further improvement making selenium an attractive high-band-gap absorber for multi-junction device applications.

## Abstract (web summary)

Wide band gap semiconductors are important for the development of tandem photovoltaics. By introducing buffer layers at the front and rear side of solar cells based on selenium; Todorov et al., reduce interface recombination losses to achieve photoconversion efficiencies of 6.5%.

## Introduction

In 1873, Willoughby Smith discovered the photoconductivity of selenium (Se)^1^. Ten years later, Charles Fritts created the first solid solar cells by coating metal foils with selenium and thin layers of gold. Puzzled by the peculiar current behavior of his cell under illumination he wondered what yet unthought-of properties this method could reveal and where its limit would be^2^. The photoelectric effect and other fundamental phenomena observed in this early semiconductor propagated through modern science and technology, leading to Einstein’s Nobel Prize work^3^, the photocopy machine, sensors, and rectifiers, among other technologies^4, 5^. Selenium research declined in the wake of the rapidly expanding silicon microelectronic industry which offered a high-quality material with a band gap (*E*_g_) more suitable for a single-junction solar cell. The development of silicon for integrated circuits provided both the science and technology that propelled silicon photovoltaic (PV) device efficiency from ∼6% in 1954 to 26.6% today^6, 7^. By contrast, Se achieved <1% in the earliest demonstrations and culminated with 5.0% in the 1980’s^2, 6^. Since then, the silicon PV industry has grown, reducing production costs and module prices, while achieving a champion module efficiency of 24.1%^8, 9^. While improved device structure, material quality and passivation have enabled increased conversion efficiency, future improvements in these areas will provide diminishing improvements as we continue to approach the practical limits for Si single junction. Tandem solar cell architectures with multiple band gaps offer the most realistic path to higher PV efficiencies surpassing the limitations of single junctions.

Until recently, multi-junction cells have been limited to low-efficiency amorphous silicon and expensive III–V semiconductor photovoltaic materials. These devices require sophisticated solar tracking concentrator systems and have been less practical for mainstream deployment in comparison with planar Si modules. For many years, the main challenge for creating efficient and low cost tandem PV devices for large-area applications has been the lack of an efficient high-*E*_g_ top cell that is process compatible for fabrication on a lower *E*_g_ absorber. Recently, metal–organic hybrid perovskite materials have reinvigorated the research of planar tandem photovoltaic devices as they offered high-efficiency solar cells with high (>1.55 eV) tunable band gaps that can be processed at low temperatures^10–13^. However, concerns about the long-term stability of these compounds, as well as their heavy metal content, have motivated the search for more stable and less toxic alternatives.

Trigonal selenium has a reported *E*_g_ between 1.83 and 2 eV depending on fabrication conditions^14, 15^_._ It can be processed at a range of temperatures below its melting point of 220 ˚C, making it an attractive candidate for top absorber in monolithic tandem or even triple-junction photovoltaic device. However, the last efficiency record of 5.0% for a Se solar cell reported in 1985 has remained unchanged for more than 30 years^16^. This champion cell utilized n-type TiO_2_, deposited on SnO_2_:F (FTO) coated glass and p-type Se followed by gold contact. This structure is similar to some modern hybrid perovskite solar cells without an advanced hole-transport layer^17^. Some recent works explored additional elements, such as mesoporous TiO_2_ in combination with poly(3-hexylthiophene-2,5-diyl) (P3HT) and poly(3,4-ethylenedioxythiophene)-poly(styrenesulfonate) (PEDOT:PSS) hole-transport layers^18, 19^. However, these approaches have not yet achieved improved efficiency values.

In this work, we demonstrate a redesigned selenium device structure with three major advances over the previous art. First, we introduced a reliable inorganic MoO_*x*_ high-work-function hole-selective layer between the selenium and the gold back contact to reduce recombination and improve collection as has been demonstrated in both CdTe solar cells as well as other inorganic and organic photovoltaics^20–22^. Second, in order to increase the benefit from the back surface field and at the same time decrease material consumption and fabrication time, we drastically reduced the thickness of the selenium absorber to only 100 nm—20 times less than the previous Se champion cell as well as typical chalcogenide absorbers such as copper indium gallium selenide (CIGS). Finally, a tunable band gap Zn_*x*_Mg_1-*x*_O (ZnMgO) buffer based on state-of-the-art developments from the field of chalcogenide photovoltaics^23^ was optimized with combinatorial studies to produce a record open-circuit voltage (*V*_OC_) of 969 mV. It is important to note that this voltage still has significant room for further improvement as it is almost 700 mV below the Shockley–Queisser (SQ) limit for the band gap of Se (1.95 eV).

## Results

### Device elements and their optimization

**Fig. 1:**
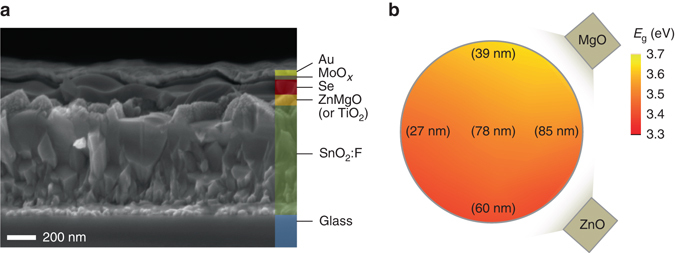
**a** Cross sectional scanning electron microscopy of the optimized device structure which consists of glass/SnO_2_:F/n-type buffer (ZnMgO or TiO_2_)/Se/MoO_*x*_/Au. Only 100-nm-thick Se absorber is used. **b** A schematic of combinatorial sputtering setup for accelerated studies on ZnMgO n-type buffer layers. A *color map* indicates the band gap (*E*_g_) of ZnMgO thin films measured by optical absorption spectra. The measured film thickness values in the map indicate a gradient of deposition rate

The fabrication of our superstrate Se solar cells is significantly simpler than other chalcogenide devices which require high temperature absorber treatments, and to some extent resembles the fabrication sequence of perovskite solar cells. The device structure in this study consists of glass/FTO/n-type buffer (ZnMgO or TiO_2_)/Se/MoO_*x*_/Au. A scanning electron microscopy (SEM) cross section of the champion device with ZnMgO buffer layer is shown in Fig. 1a.

**Fig. 2:**
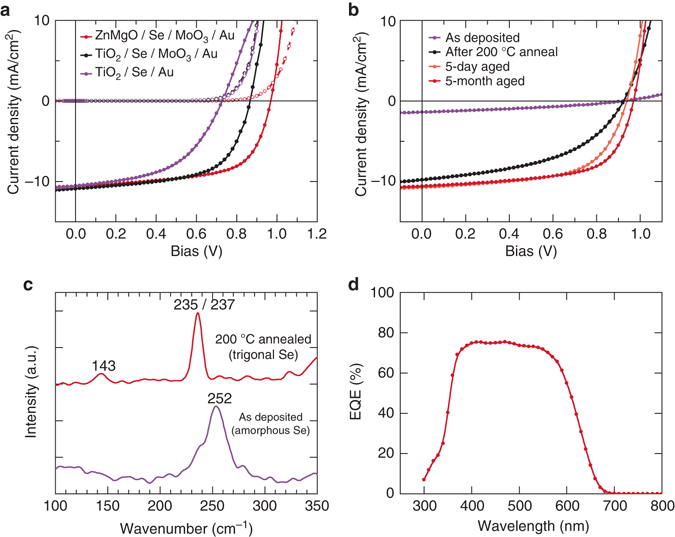
**a** As-measured current–voltage (*J*–*V*) characteristics of Se solar cell devices under dark (*dashed lines*) and illuminated (*solid lines*) condition, showing the effect of buffer and hole-transport layer. ZnMgO/Se/MoO_*x*_/Au, TiO_2_/Se/MoO_*x*_/Au, and TiO_2_/Se/Au devices are shown in *red*, *black*, and *purple*, respectively. **b** Illuminated *J*–*V* plots of ZnMgO/Se/MoO_*x*_/Au devices with as-deposited Se, annealed at 200 ˚C, 5-day aged after annealing, and 5-month aged after annealing Se are shown in *purple*, *black*, *orange* and *red*. **c** Raman spectra of as-deposited Se (*purple*) and annealed Se at 200 °C (*red*). The annealed Se shows convoluted peaks at 235 and 237 cm^−1^. **d** External quantum efficiency spectrum of a ZnMgO/Se/MoO_*x*_/Au device

Initial experiments utilized dense TiO_2_ electron-selective layers deposited by spray pyrolysis on commercial FTO glass substrates, as is commonly done for dye-sensitized and perovskite solar cells^24^. This layer served for the initial device structure optimization, including the MoO_*x*_ back contact layer that produced our first efficiency improvement (Fig. 2a).

The Se absorber was deposited by thermal evaporation which does not require high vacuum as Se evaporates readily at low temperature. The as-deposited selenium is red with a *E*_g_ of 2.17 eV determined by UV–vis absorption spectroscopy and comprises mainly the amorphous phase. This phase gave very low efficiency (Fig. 2b), exhibited high resistivity and had poor collection at long wavelengths as evidenced by external quantum efficiency (EQE) suggesting significant recombination in the amorphous phase. Subsequent annealing at 200 ˚C for 2 min produced dark brown to gray samples correlated with conversion to trigonal selenium phase confirmed by Raman spectroscopy as shown in Fig. 2c. This also decreased the effective band gap of the absorber to about 1.95 ± 0.04 eV as determined from the Tauc plot in close agreement with the EQE derivative method using inflection of the EQE curve near the band edge (Fig. 2d). As with earlier works, we found that device performance increased with temperature up to about 200 °C^6^_,_ above which severe device deterioration occurred linked to loss of a conformal Se layer coverage due surface de-wetting near the Se melting point. To improve Se adhesion and uniformity, as has been previously demonstrated^6^, a tellurium layer on the order of 1 nm was deposited on the buffer layer prior to Se deposition. Te adhesion layer was critical for obtaining functional devices as otherwise the Se layer becomes porous during annealing leading to shunting. Larger quantities of Te are expected to reduce the *E*_g_ and therefore could increase the efficiency in the typical AM1.5 spectrum, but were found to decrease performance in our testing due to lower shunt resistance which requires further investigation. Hall measurements were performed using a rotating parallel dipole line AC Hall system^25^ on a 105 nm Se/ZnMgO/glass sample annealed at 200 °C which yields a low carrier density of 2.8 × 10^12^ cm^−3^ and mobility of 0.46 cm^2^ V^−1^ s^−1^. This should serve as a good estimate for the Se film characteristics as the standalone ZnMgO sheet resistance was too high to measure in our setup (∼ > 1 TΩ per square). Compared to most PV absorbers the carrier density and mobility in these Se films is quite low^26^. Increasing the carrier density without significantly increasing recombination may be an effective means for future voltage improvements.

Functional devices were obtained with a selenium thickness range of 25–200 nm with highest performance found at about 100 nm. While a thicker absorber is expected to increase the number of photons absorbed, particularly for wavelengths near the *E*_g_, this is counter acted by decreasing carrier collection for thicker samples due to the smaller effect of the back surface field, and the increasing resistance for thicker absorbers which further reduces the fill factor. At the Se band gap, 1.95 eV, the transmission through the FTO + 100 nm Se layer is only 16%, suggesting that this thickness is sufficient to absorb most of the available photons when coupled with the additional benefit of reflective metal back contact. Remarkably, devices as thin as 25 nm were able to produce efficiencies in excess of 2.7%. The transmission data for a solar cell fabricated following the same procedure of the record device without a gold back contact is shown in Supplementary Fig. 1.

The hole-selective MoO_*x*_ back layer (10–30 nm thick) and Au contacts (50–100 nm thick) were deposited by thermal evaporation. No additional flow of oxygen was used during the evaporation from MoO_3_ powder, and therefore it is expected that oxygen vacancies in the deposited material will be present. This would result in modified work function of MoO_*x*_; however, this can vary with subsequent exposure to air^27^ and requires further investigation. We found an optimal MoO_*x*_ thickness around 20 nm. As can be seen in Fig. 2, the introduction of MoO_*x*_ produced an improvement in both the fill factor from 50.5 to 60.6% and the *V*_OC_ from 728 to 866 mV. It is important to note that all cell parameters significantly improve in the first 5 days after fabrication followed by slow increase measured up to 5 months (Fig. 2b). One possible explanation for this performance improvement is the change of the properties of the MoO_*x*_ upon air exposure which has been linked to aging performance improvements in other systems using MoO_*x*_ layers^27^.

The wide band gap of the Se absorber puts additional constraints on the choice of optimal electron-selective contact or n-type buffer material. This layer must repel holes, which can be achieved by inducing downward band bending of the valence band while also allowing electrons to pass. A slight spike in the conduction band alignment can further create a slight barrier to recombination at the interface, while still low enough to allow current flow. The desired type-II band alignment can be difficult to achieve due to the difficulty finding buffer materials with conduction band positions above that of Se due to the high-band gap^28^.

Tunable buffers offer an opportunity to optimize valence and conduction band alignment with the absorber. One such buffer, ZnMgO, is particularly attractive since its conduction band position can be tuned through the introduction of varying Mg doping. This material has been successfully used for improving the band alignment in CIGS- and polymer-based PV devices^29, 30^. The *E*_g_ of ZnO and MgO are 3.3 and 7.8 eV, respectively. ZnO has a conduction band edge below that of Se, so the addition of low concentrations of Mg is expected to move the conduction edge upward, therefore reducing the magnitude of the cliff. This buffer/absorber interface also serves as electron-selective contact and the band bending helps repel the holes from the interface thus minimizing the surface recombination.

**Fig. 3:**
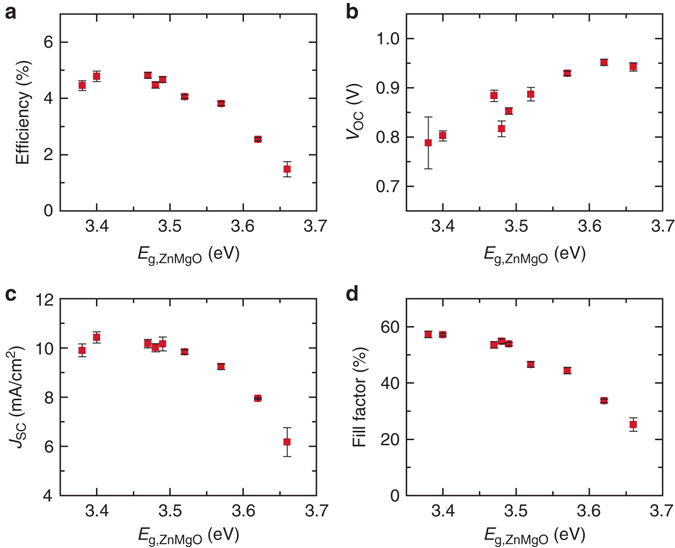
Effects of band gap (*E*_g_) of ZnMgO buffer layer on the photovoltaic characteristics. **a** Power conversion efficiency, **b** Open-circuit voltage (*V*_OC_), **c** Sort circuit current (*J*_SC_), and **d** fill factor of the ZnMgO/Se/MoO_*x*_/Au devices prepared by the combinatorial deposition process are plotted against measured *E*_g_ of ZnMgO layer. *Square symbols* and *error bars* indicate average values and standard deviations, respectively

We used a simple confocal sputtering setup^31^ for accelerated combinatorial studies of ZnMgO buffer devices with different [Zn]:[Mg] ratios as well as buffer thicknesses based on co-sputtering of mixed composition from separate ZnO and MgO sources, as depicted in Fig. 1b. The decreasing deposition rate with the distance from each source produced transverse gradients in *E*_g_ of ZnMgO and total thickness across an 8 inch diameter sample area. Figure 3 shows the device parameters obtained at indexed coordinates showed clear correlation between the *E*_g_ of the buffer layer and performance. While the *V*_OC_ continues to increase with the *E*_g_ of ZnMgO, a trade-off is observed with increased series resistance resulting in a lower fill factor. Current-density vs. voltage characteristics of representative devices with various ZnMgO buffer layers and averaged series resistance values under 1-Sun illumination are shown in Supplementary Fig. 2. Optimal buffer thickness was determined to be in the range of 60–85 nm (Supplementary Fig. 3) with an optimal band gap of 3.4 eV that corresponds to approximate composition of Mg/(Zn + Mg) = 0.1 determined by deposition rate calibration in close agreement with literature data on the ZnMgO system optical properties^23^. A standard sputtering process with sample rotation was then developed to produce baseline buffer layers with noted optimal composition and thickness for further studies, enabling the current record-performance ZnMgO devices.

**Fig. 4:**
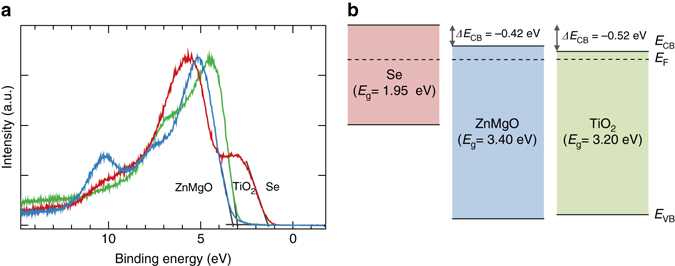
**a** Femtosecond ultraviolet photoelectron spectroscopy (fs-UPS) spectra of Se (*red*), TiO_2_ (*green*), and ZnMgO (*blue*) under flat-band conditions. **b** Valence band and conduction band lineups extracted from the fs-UPS and optical absorption spectra

Femtosecond pump-probe ultraviolet photoelectron spectroscopy (fs-UPS) was employed to study the band alignments between Se and the buffers^32, 33^. This technique has been described elsewhere (put references here). Briefly, correlated 40 fs pump (3.1 eV) and probe (26.35 eV) pulses were used to carry out UPS on Se and ZnMgO/Se and TiO_2_/Se surfaces under flat-band conditions. Band bending in the Se was extracted by comparing the energetic shifts of pumped and unpumped spectra. Figure 4a shows an overlay of the UPS spectra for Se and ZnMgO where the valence band maximum (*E*_VB_) relative to the Fermi level (*E*_F_) can be found from the well-established method of extrapolating the valence density of states to zero intensity. The Fermi level was determined in the standard fashion for UPS measurements by measurement of a clean metal surface in electrical contact with the sample. The valence band maximum for the clean Se surface (seen in red) is found to be 1.28 eV below *E*_F_, shown as 0 eV. A thin ZnMgO layer was deposited on top of the Se and is also shown in the spectrum in blue. The valence band offset for this heterojunction is 1.87 eV.

Once the valence band offset was determined using fs-UPS, the conduction band offset is calculated from the measurement of the band gaps of both the Se (1.95 eV) and ZnMgO (3.4 eV determined from the Tauc plot of the optical absorption) and adding these values to the valence band maxima of Se and ZnMgO respectively. The determination of the valence band maximum was accurate to within ± 0.05 eV. The resulting measurement confirms a cliff-like band offset of 0.42 eV. Further increases in [Mg] should increase in the conduction band minimum of the ZnMgO and reduce the cliff offset which would further improve the *V*_OC_ of the ZnMgO/Se devices; however, this produced an increased resistivity and a drop in the fill factor, resulting in lower efficiency in our devices. A useful comparison can be made with TiO_2_ as a buffer material. This is shown on the right side of Fig. 4b schematic. The TiO_2_ valence band maximum is located 3.05 eV below *E*_F_ and with a *E*_g_ of 3.2 eV this would locate the conduction band minimum 0.52 eV below that of Se, producing an even larger cliff offset than the ZnMgO. This is consistent with the lower *V*_OC_ and lower performance of devices fabricated with TiO_2_ as a buffer material.

### Device characterization

Table 1 shows a comparison between the three device structures from this work: a FTO/TiO_2_/Se/Au device—similar to the previous 5.0% champion device^6^, an improved high-work-function back contact FTO/TiO_2_/Se/MoO_*x*_/Au device, and finally an optimized n-type ZnMgO buffer FTO/ZnMgO/Se/MoO_*x*_/Au device. Progressive improvement in performance is observed first with the introduction of MoO_*x*_, resulting in improved fill factor from 50.5 to 60.6% and *V*_OC_ enhancement from 728 to 866 mV. The introduction of higher band gap ZnMgO produced additional 100 mV increase in *V*_OC_ and 3% increase in fill factor.

**Table 1:** Top performing Se devices

Device	Efficiency (%)	Fill factor (%)	*V*_OC_ (V)	*J*_SC_ (mA cm^−2^)	*n*_1_	*J*_01_ (A cm^−2^)	*R*_S_ (Ω cm^2^)	*G*_sh_ (S cm^−2^)
ZnMgO/Se/MoO_*x*_/Au	6.51	63.4	0.969	10.6	2.78	1.09 × 10^−8^	0.213	1.09 × 10^−8^
					(2.23)	(1.47 × 10^−7^)	(0.655)	(1.58 × 10^−11^)
TiO_2_/Se/MoO_*x*_/Au	5.73	60.6	0.866	10.9	3.14	1.94 × 10^−8^	0.828	2.19 × 10^−8^
					(1.93)	(1.54 × 10^−8^)	(1.18)	(1.06 × 10^−5^)
TiO_2_/Se/Au	3.88	50.5	0.728	10.5	4.00	7.48 × 10^−6^	6.255	2.63 × 10^−3^
					(1.95)	(1.59 × 10^−10^)	(1.15)	(8.83 × 10^−6^)
SQ limit (*E*_g_ = 1.95 eV)	23.9	92.1	1.65	15.7	1	2.99 × 10^−30^	0	0

Statistics on device variation within a batch is shown in the SI. The single diode parameters in illuminated and dark conditions are also presented: ideality factor (*n*_1_), dark reverse saturation current (*J*_01_), series resistance (*R*_S_) and shunt conductance (*G*_sh_). The values with parentheses are extracted from dark current–voltage (*J*–*V*) measurement. Shockley–Queisser (SQ) limit calculated from refs. ^36, 37^

The best performing FTO/ZMO/Se/MoO_*x*_/Au device had approximate efficiency of 6.5% with a *V*_OC_ of 969 mV, fill factor of 63.4% and *J*_SC_ of 10.6 mA cm^−2^. The *J*_SC_ values were calibrated with EQE measurement (Fig. 2d) in order to eliminate any error due to solar simulator spectral mismatch which can have outsize effects on high-*E*_g_ solar cells. This should also eliminate internal reflection from outside the cell area known to be a common issue in superstrate solar cells^34^. The average efficiency of eight cells from the same batch was 6.2% with a standard deviation of 0.28%. The feasibility of larger cells, was also demonstrated with cell areas ∼¼ cm^2^. These cells demonstrated generally comparable performance to the small dot cells which suggests reasonable device uniformity is achieved. The device statistics comparing small and large cells is summarized in Supplementary Table 1 and full device data is shown in Supplementary Fig. 4. Many Se devices exhibit slight hysteresis resulting in about 6–7% difference in the maximum power point depending on the scan direction (Supplementary Fig. 5). Slower scans also produce decrease in efficiency but proper interpretation would require modified constant-temperature measurement setup to mitigate the concurrent sample warming. Hysteresis is not common in chalcogenide absorbers and its origin as well as ways to address it are under investigation.

Further gains in *J*_sc_ should be readily achievable with improving transparent conductive oxide (TCO) and adding an appropriate anti-reflective coating. In particular the FTO used in this study which has 80–82% visible transparency specification was measured via UV–vis to transmit only 72% of the above the band-gap photons in the AM1.5 G spectrum (280–635 nm). While this transmittance measurement may overstate the absorption losses in the system, it still can be surmised to account for a large portion of the EQE loss at lower wavelengths.

We extracted the one-diode parameters (ideality factor *n*_1_, reverse saturation current *J*_0_, series resistance *R*_S_ and shunt conductance *G*_sh_) to elucidate the loss mechanisms in this device. We used curve fitting technique based on Lambert-W function as described in ref. ^35^ which yields a very good fit. The theoretical SQ limit for a solar cell with 1.95 eV *E*_g_ absorber solar are shown at the bottom row of Table 1. These limits are calculated under 1-Sun (AM1.5 G, 100 mW cm^−2^) with assumptions described in refs. ^36, 37^. We observe that even though the *V*_OC_ for our champion cell is high, it is significantly lower than its theoretical SQ-limit *V*_OC_ of 1650 mV. This *V*_OC_ deficit (i.e., *E*_g_/*q*-*V*_OC_, where *q* is the electron charge) of 981 mV shows there is significant potential for improvement. The second problem is the fill-factor (FF) which is about two thirds of the value achievable in the SQ limit. This is mostly attributed to the high ideality factor (*n*_1_) and high *G*_sh_ under light. The high *n*_1,_ larger than 2, suggests several possible factors such as severe recombination in the space charge region, buffer/absorber interface recombination commonly found in heterostructure or tunneling enhanced recombination^38^. Note that the shunt conductance in the dark (*G*_sh,Dark_ = 1.58 × 10^−11^ S cm^−2^) is very low indicating no physical shunt or leakage problem across the device, however the light shunt conductance (*G*_sh,Light_) is higher than the dark indicating voltage-dependent collection-efficiency problem^37^. This problem occurs due to short minority carrier collection length which is caused by high recombination and resulting low minority carrier lifetimes. In this situation, increasing the forward bias will reduce the space charge region and thus the carrier collection significantly—as the collection due to diffusion is minimal. This results in a positive slope near *V* = 0 V or higher shunt conductance under light (*G*_sh,Light_). The series resistance under light is small (<1 Ω cm^2^) indicating low resistance from top and bottom contacts to the device.

**Fig. 5:**
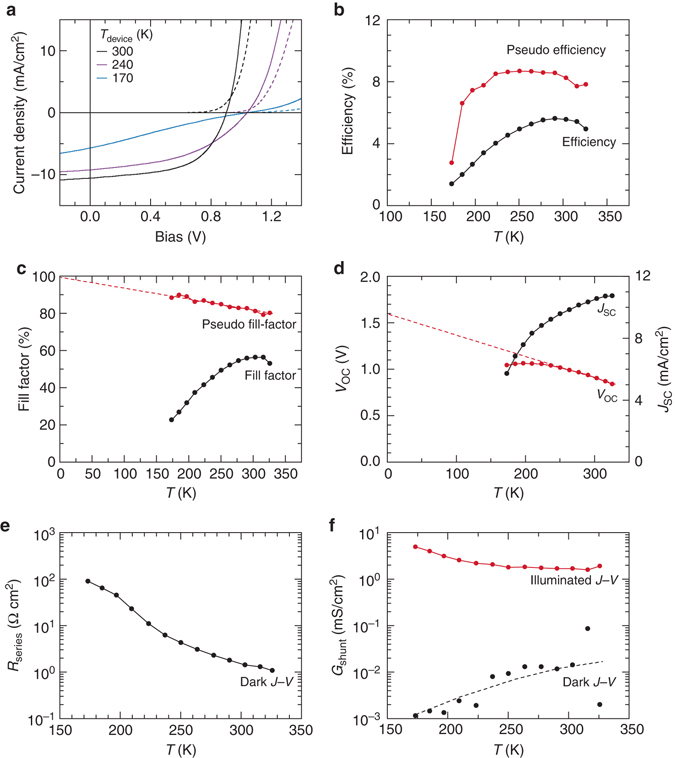
Temperature-dependent *J*–*V* characteristics of the SnO_2_:F/ZnMgO/Se/MoO_*x*_/Au device. **a** Current–voltage (*J*–*V*) plots under 1-Sun illuminated (*solid line*) and dark (*dashed line*) conditions measured at the device temperature of 300 °K (*black*), 240 °K (*purple*), and 170 °K (*blue*). **b** Efficiency (*black*) and pseudo-efficiency (*red*) plots. **c** Fill factor (*black*) and pseudo-fill-factor (*red*). The pseudo-efficiency and pseudo-fill-factor were extracted from the short circuit current (*J*_SC_)—open-circuit voltage (*V*_OC_) measurements under various illumination intensities. **d** *J*_SC_ (*black*) and *V*_OC_ (*red*). **e** Series resistance (*R*_S_) extracted from dark *J*–*V* measurements. **f** Shunt conductance (*G*_sh_) extracted from the illuminated (*red*) and dark (*black*) *J–V* measurements

We performed temperature-dependent study of a device similar to the champion Se device (ZnMgO/Se/MoO_*x*_/Au) on a liquid-nitrogen cooled cryostat under 1-Sun illumination. The efficiency 5.6% at 25 °C of this device as measured in the cryostat station is about 1% (absolute) lower than typical probe stage readings with a lower FF due to a non-ideal contact in our cryostat setup. The efficiency and pseudo-efficiency (the expected in the absence of series resistance, efficiency derived from the *J*_SC_–*V*_OC_ measurement under various illumination intensities) is plotted in Fig. 5. We observe that as the temperature decreases, the efficiency slightly increases and then drops monotonically. The drop in efficiency begins at a relatively high temperature (280 ˚K). This behavior is unlike most other high performance solar cells where higher efficiency occurs down to lower temperatures (100 ˚K) before the efficiency crashes^39^. Among possible factor for this behavior is the carrier freeze-out effect that occurs in materials with very deep impurity level and increasing blocking transport behavior at the interfaces (e.g., buffer/absorber or absorber/back contact) which easily occurs in low carrier density material. The dominant contributor to the temperature-dependent efficiency behavior is the fill-factor behavior vs. temperature as will be discussed later.

Another important observation can be obtained from the *V*_OC_ and *J*_SC_ plot in Fig. 5b. The *V*_OC_ plot shows the usual initial increase at low temperature, which is expected as recombination is reduced at lower temperatures, decreasing the dark saturation current. However below 220 ˚K the *V*_OC_ starts to level off. This behavior has been observed in samples with deficient carriers as the device can no longer sustain high *V*_OC_^40, 41^. This fact gives another evidence for the freeze-out effect in this device at low temperature. The 0 ˚K intercept of the *V*_OC_ points to 1.6 eV is consistent with the cliff expected at the ZnMgO/Se interface from fs-UPS measurement, still far below the band gap of 1.95 eV. The measurements suggest that interface recombination is a pre-dominant factor for limiting *V*_OC_ in this device. The *J*_SC_ also shows monotonically decreasing behavior at low temperature. Normally in an ideal solar cell, *J*_SC_ only changes a little with respect to temperature. The marked decrease in the *J*_SC_ here is due to the significant increase in series resistance (*R*_S_) at low temperatures that distort the *J*–*V* curve and lower its value at low temperature. This occurs approximately when *R*_S_ is larger than *V*_oc_/*J*_sc_. The fill factor vs. temperature profile is determinant factor in the efficiency behavior, i.e., it increases slightly but quenches at low temperature. Specifically, the quench at low temperature is attributed to the increase in bias dependent collection limitation (responsible for very high shunt conductance G_sh,light_) and increase in series resistance *R*_S_ as shown in Fig. 5e, f. The dark *R*_S_ shows increasing behavior at low temperature that could originate from the carrier freeze-out effect of the absorber or increasing non-ohmic contact between the absorber and back contact. Finally, in Fig. 5f, we show that *G*_sh,Light_ is always larger than the *G*_sh,Dark_ due to the voltage dependent collection efficiency problem as described earlier.

## Discussion

We have demonstrated a substantial efficiency improvement for Se photovoltaics, 30 years after the last efficiency milestone was set, and further outlined key areas for future improvements. Three areas of focus may offer particularly large improvements. First, optimizing the buffer layer to reduce the cliff at interface should significantly improve *V*_OC_ as well as fill factor. Second, increasing lifetime and/or increasing the depletion width should reduce the voltage-dependent collection thus improving FF and further improving *V*_OC_. Lastly, higher-transmission TCO, an anti-reflective coating, and improved reflection of the back contact coupled with thickness re-optimization is expected to boost *J*_SC_ by increasing absorbed photons. Optimization of TCO alone may allow >10% increase in *J*_SC_ due to the high absorbance at low wavelengths. Additional strategies such as increasing carrier density of the Se absorber through doping may offer a further means to improve the *V*_OC_.

In addition to higher performance potential, other qualities make Se an attractive alternative candidate for a high-band-gap absorber. For practical applications, a simple and inexpensive fabrication process, lack of highly toxic elements such as Cd and Pb, and stability upon prolonged storage and air exposure for these devices (for the duration of our testing) are significant advantages. More detailed stability testing under real operation condition is required. In addition, due to the ultra-thin layers, material usage is minimal and therefore material availability and raw material costs are not practical concerns. The potential to scale production to large-scale manufacturing is aided by fast and low-temperature deposition methods which also enable the use of plastic and flexible substrates or roll-to-roll fabrication. The high *E*_g_ makes Se attractive as a top cell in multi-junction devices and the low processing temperature processing could allow drop-in compatibility with crystalline Si or CIGS thin film manufacturing. In addition, the high-band gap shows strong match as a single junction for the indoor lightning spectrum (increased blue range)^6^, coupled with high *V*_OC_ and simple fabrication offers a strong fit for consumer electronics power solutions including autonomous sensors, wearable and internet-of-things devices^42^. After more than three decades with no improvements and little research attention, selenium solar cells may deserve a second look.

## Methods

### Solar cell fabrication

Unless otherwise specified all materials were purchased from Sigma Aldrich. Fluorine-doped Tin Oxide glass (7 Ω per square, 80–82% visible light transmittance) was washed consecutively with acetone, isopropanol and ammonium hydroxide. TiO_2_ buffers with thickness about 30–50 nm were air-sprayed from a 10 vol% each: titanium di-isopropoxide and acetylacetone solution in ethanol on a substrate preheated at 500 ˚C followed by annealing at 540 ˚C for 10 min. ZnMgO buffer layers were deposited by the simultaneous radio-frequency sputtering from ZnO (99.999%) and MgO (99.95%) ceramic targets (Kurt Lesker) mounted on adjacent guns in a 5-source CMS-18 sputtering system (Kurt J. Lesker). An 8″ sample holder was used. For combinatorial studies the sample position was indexed and no rotation was used. For baseline process rotation was used and the deposition rate was ∼0.5 nm min^−1^. No temperature control was used for the samples during sputtering and evaporation. All thermal evaporations were done from resistively heated alumina crucibles. Te and Se films were deposited by using a custom-built 8″ diameter, 12″ throwing distance thermal evaporation chamber under a pressure of 5 × 10^−6^ Torr and approximate rate of 20 nm min^−1^. Final devices were fabricated by thermal evaporation of MoO_3_ (99.5% Alfa Aesar, 4 nm min^−1^) and Au (99.99%, Alfa Aesar, 10 nm min^−1^), on the top of Se using dot masks. Deposition rates were controlled with INFICON quartz crystal monitor and manual power adjustment. All annealing was performed by placing samples on a pre-heated aluminum hot plate for 2 min and temperature calibration was performed with a thermocouple glued to a dummy sample. Cell area was confirmed via microscope and LBIC (Light-beam induced current) measurement to be equal to back contact mask size of 2.27 mm^2^.

### Characterization

*J*–*V* characteristics of the devices were measured by using a Keithley 2400 source-meter. A 6 × 6 in^2^ beam solar simulator equipped with a 1000 W Xe arc lamp with an AM1.5 G filter (Newport). In order to work in superstrate configuration we used double perpendicular aluminum-coated mirror reflector adapter calibrated to 1-Sun illumination condition (100 mW cm^-2^) with n NREL-traceable silicon reference cell with a BK-7 window was used to calibrate the illumination intensity. Despite calibrated light intensity, a spectral mismatch using the superstrate test setup was noted which resulted in a slight increase in blue light which can results in overstated *J*_SC_ for high-*E*_g_ absorbers. To correct for this, as well as possible internal glass reflection in superstrate configuration the current values in this paper were adjusted down to match a calibrated EQE value. Standard voltage scans were done at a rate of 1 V s^−1^ backward direction in air at room temperature.

The temperature-dependent *J*–*V* measurement was performed in a nitrogen-filled chamber using a liquid-nitrogen cryostat with IBM-PVX software system^43^ with rotating neutral density filter for integrated *J*_SC_–*V*_OC_ measurement for pseudo-efficiency and pseudo-fill-factor measurement. Two temperature sensors are used: one on the cold stage for the closed-loop temperature control and second clamped on the device to sense the device temperature. The one-diode model parameters (ideality factor *n*_1_, ideality factor *J*_0_, series resistance *R*_S_ and shunt conductance *G*_sh_) are determined using fitting method based on Lambert-W function^35^. The EQE of the device was measured by using a QEX10 (PV Measurements) calibrated with a NIST-certified Si photodiode. Optical absorption spectra were measured by using a Lambda 950 UV–vis spectrometer (Perkin Elmer). Raman spectra were measured ex situ with a 15 mW 532 nm laser on a Horiba LabRAM system using a ×10 confocal lens and an 1800 g mm^−1^ diffraction grating. A neutral density filter of ND 4.0 was inserted to mitigate photo-bleaching of the samples. A long exposure time of 100 s was used with the lower magnification to improve signal to noise ratio. Data were smoothed with a five-point moving window FFT filter. For fs-UPS measurements, a single 800 nm pulse from an amplified fs laser is split into synchronized pump and probe legs. The 800 nm pump pulse is frequency doubled to 400 nm (3.1 eV per photon) and used to excite a dense electron hole population within the Se absorber, flattening the bands. The 800 nm probe pulse is converted to 26.35 eV via high harmonic generation in a gas and is used to generate the UPS spectra. In this manner, the valence band edges can be determined under flat band conditions and accurate offsets can be determined.

### Data availability

The data that support the findings of this study are available from the corresponding author upon request.

## Electronic supplementary material

Supplementary Information
