# Supplementary material for: Ultrathin high band gap solar cells with improved efficiencies from the world’s oldest photovoltaic material
Source: Nat Commun. 2017 Sep 25;8:682. doi: 10.1038/s41467-017-00582-9 (PMC5613033; doi:10.1038/s41467-017-00582-9)
Supplement: Supplementary file 1 — Supplementary Information [file 41467_2017_582_MOESM1_ESM.pdf]

Description of Supplementary Files

File Name: Supplementary Information
Description: Supplementary Figures and Supplementary Table

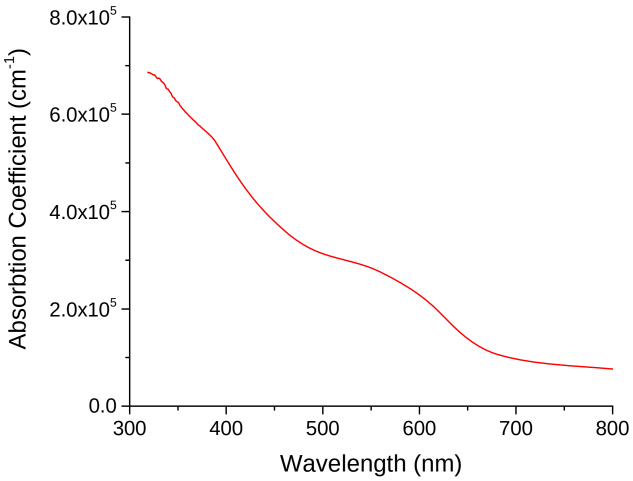

Supplementary Figure 1. Absorption data for Se solar cell. The SnO:F (FTO) substrate is used for zero absorbance baseline.

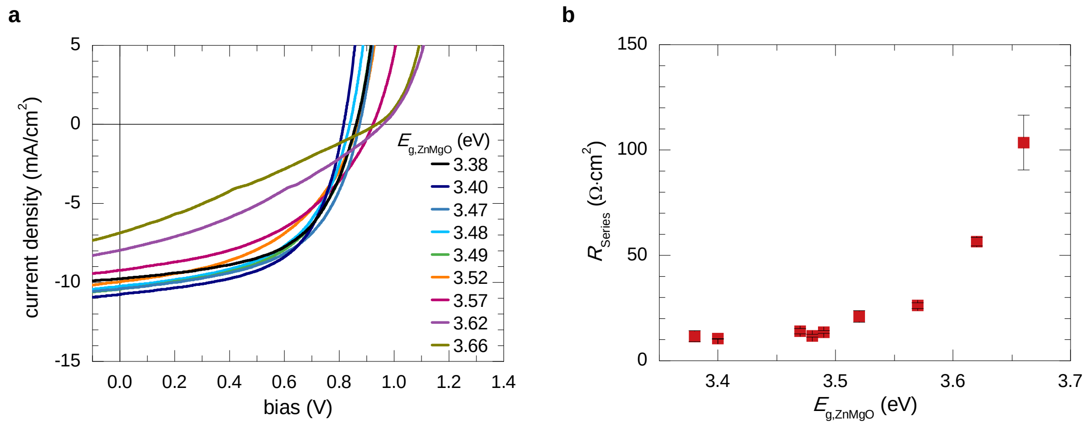

Supplementary Figure 2. (a) Current-density *vs*. voltage characteristics of representative devices with various ZnMgO buffer layers and (b) averaged series resistance values under 1-Sun illumination.

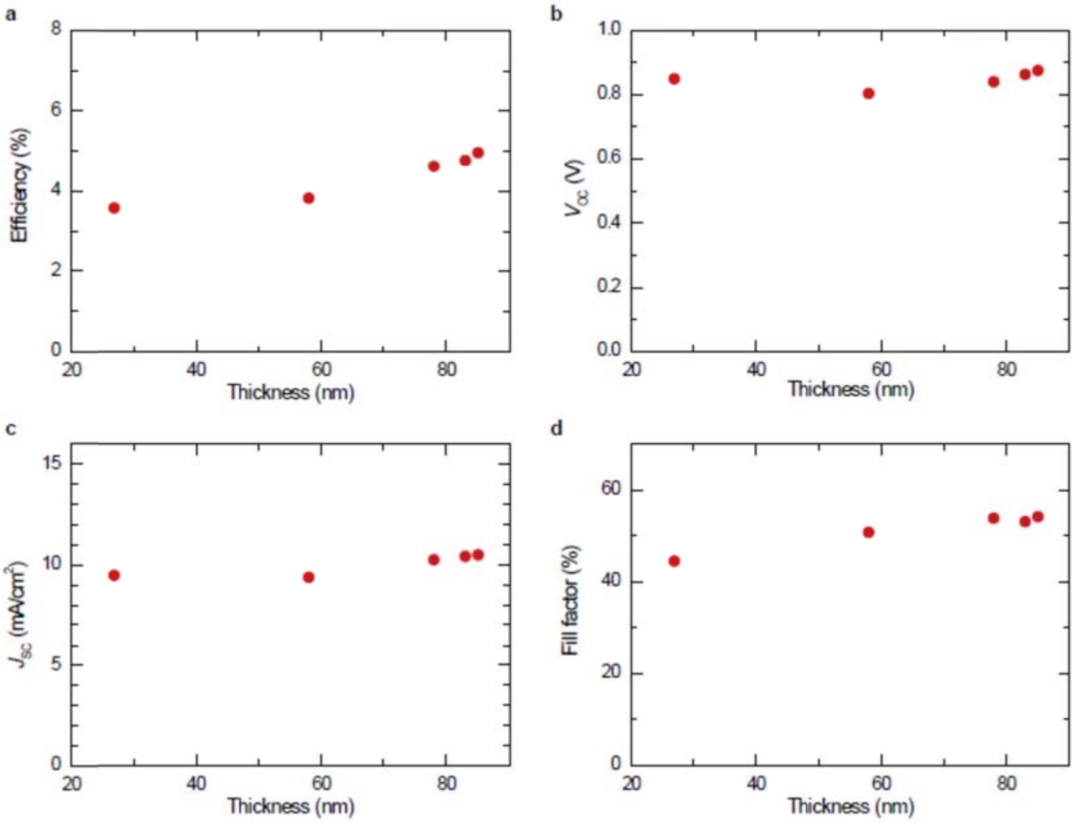

Supplementary Figure 3. Effects of ZnMgO buffer layer thickness on the photovoltaic (PV) characteristics (as-measured data). Devices with band gap (E_g) of ZnMgO between 3.45 and 3.5 eV are plotted. (a) Power conversion efficiency, (b) Open circuit foltage (V_{OC}), (c) Short circuit current (J_{SC}), and (d) fill-factor (FF) of the ZnMgO/Se/MoO_x/Au devices prepared by the combinatorial deposition process are plotted against measured thickness of ZnMgO layer.

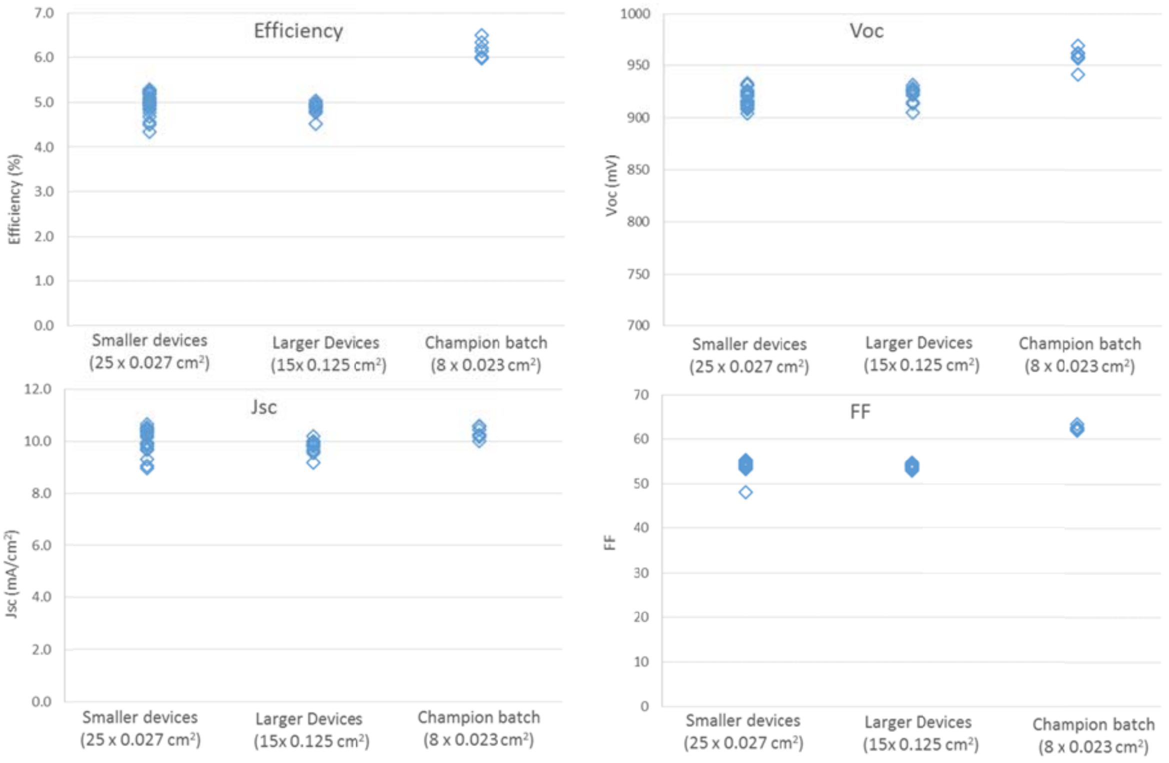

Supplementary Figure 4. Statistical data for small and large cells from a single batch showing general process uniformity and similar efficiency for small and large cells. Area correction was not performed on individual devices which leads to larger scatter in small area device currents. Champion batch with optimized layers shown for comparison.

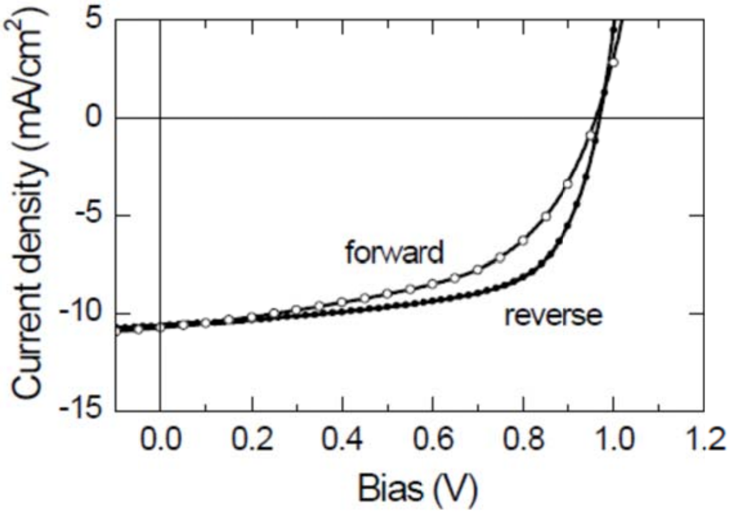

Supplementary Figure 5. Hysteretic behavior of a representative device with ZnMgO buffer layer resulting in about 7% difference of the maximum power point depending on the scanning direction (scan rate 1V/s).

Supplementary Table 1. Statistical analysis of data from Supplementary Figure 2.

		Efficiency (%)	Voc (mV)	Jsc (mA/cm^2)	FF (%)
Larger Devices (15 x 0.125 cm^2)	Median	4.89	925.2	9.84	54.05
	Std. Dev.	0.36	25.5	0.24	2.65
	% Dev	7%	3%	2%	5%
Smaller Devices (25 x 0.027 cm^2)	Median	5.0	917.1	10.2	54.2
	Std. Dev.	0.25	8.4	0.46	1.40
	% Dev	5%	1%	4%	3%
